# Incidence of cervical intraepithelial neoplasia and cervical cancer in transmasculine and gender diverse individuals using testosterone: a retrospective, single-centre cohort study

**DOI:** 10.1016/j.eclinm.2024.103037

**Published:** 2025-01-13

**Authors:** Asra Vestering, Wouter L.J. van Vugt, Alison M. Berner, Malou L.H. Snijders, Martin den Heijer, Freek A. Groenman, Judith A.F. Huirne, Chantal M. Wiepjes, Norah M. van Mello

**Affiliations:** a Department of Obstetrics and Gynaecology, Amsterdam UMC, Location Vrije Universiteit Amsterdam, Amsterdam, the Netherlands; b Amsterdam Reproduction and Development Research Institute, Amsterdam, the Netherlands; c Centre of Expertise on Gender Dysphoria, Amsterdam University Medical Centre, Amsterdam, the Netherlands; d Barts Cancer Institute, Queen Mary University of London, Londen, United Kingdom; e Gender Identity Clinic, Tavistock and Portman NHS Foundation Trust, London, United Kingdom; f Department of Pathology, Amsterdam UMC, University of Amsterdam, Amsterdam, the Netherlands; g Department of Endocrinology, Amsterdam UMC, VU University Medical Centre, Amsterdam, the Netherlands

**Keywords:** Transmasculine and gender diverse, Cervical cancer, Cervical dysplasia, HPV, Testosterone

## Abstract

**Background:**

The number of transmasculine and gender diverse (TMGD) individuals who retain their uterus or postpone surgery while using testosterone is increasing. However, the influence of exogenous testosterone on the risk of cervical cancer remains unclear. This study aims to assess the risk of cervical cancer and intraepithelial neoplasia in TMGD individuals undergoing testosterone treatment.

**Methods:**

This retrospective, cohort study was conducted at the Amsterdam University Medical Centre in the Netherlands, included transmasculine and gender diverse (TMGD) individuals receiving testosterone at our clinic between February 17, 1972 and December 3, 2018. Data from medical records were linked to the national pathology database to acquire diagnoses related to cervical cancer or cervical intraepithelial neoplasia (CIN). Individuals assigned female at birth who received testosterone were included, excluding those last seen before 1991. Lesions ≥ CIN2 were classified as “high grade”, considering their increased cancer progression risk. Based on observed and expected cases, age-adjusted standardised incidence ratios (SIR) were calculated to assess relative risk compared to cisgender women.

**Findings:**

The cohort comprised 2095 TMGD individuals; 1200 participants underwent hysterectomy, and cervical biopsies obtained from seven patients. Median testosterone exposure time was 1.7 years (IQR 1.3–2.5). No cervical cancer cases were observed (0.30 (95% CI 0–1.4) expected). Five cases of ≥CIN2 (0.002%) were observed, versus 9.5 expected (SIR 0.53 (95% CI 0.19–1.17).

**Interpretation:**

In this large cohort with several years of testosterone exposure we did not observe any cervical cancer, nor did we observe an increased risk of ≥CIN2. These findings should be interpreted with caution, as the relatively short median time of follow-up and lack of data on HPV infection prevalence and cervical screening may introduce bias. Longer follow-up studies incorporating this information are needed.

**Funding:**

None.

**Research in context**

**Evidence before this study**

We conducted a comprehensive search of the PubMed database covering the existing literature until January 03, 2024. Search terms included transmasculine individuals, gender dysphoria, masculinising hormone therapy, testosterone, androgen, cervix or cervical dysplasia, along with synonyms. Studies were included if they focused on the risk of cervical cancer or intraepithelial neoplasia in transmasculine and gender-diverse individuals undergoing testosterone treatment, without restrictions on publication language. We also reviewed references listed in the identified papers. Our search returned 38 results. Previous studies have predominantly focused on barriers specific to transmasculine and gender diverse individuals regarding appropriate cervical cancer screening, leading to higher rates of inadequate results and lower rates of regular screening. Only a limited number of individual cases of cervical cancer in transmasculine and gender-diverse individuals using testosterone were reported, as well as small-scale studies describing the incidence of cervical intraepithelial neoplasia. No studies were found that provided data enabling the assessment of the risk of cancer or intraepithelial neoplasia in this population compared with the cisgender female population.

**Added value of this study**

As the number of transmasculine and gender diverse individuals using testosterone while retaining their uterus or postponing surgery increases, risk-assessment of cancer or intraepithelial neoplasia is becoming increasingly relevant. To the best of our knowledge, our cohort, comprising 2095 transmasculine and gender diverse individuals with several years of testosterone exposure, represents the first large-scale study to comprehensively investigate incidences within this specific population. No observations of cervical cancer were observed, and the age-adjusted incidence of high-grade cervical intraepithelial neoplasia was lower than expected based on national incidences in cisgender women.

**Implications of all the available evidence**

Our findings, including the absence of cervical cancer cases and no increased risk of high-grade cervical intraepithelial neoplasia compared to cisgender women, provide reassurance regarding the safety of testosterone treatment in transmasculine and gender diverse individuals. However, potential effects could be masked by the young age at hysterectomy and the relatively short duration of testosterone exposure observed in our study.

Future research should be prospective in nature and include longer follow-up periods to capture potential long-term effects. Additionally, it should account for potential risk factors such as sexual behaviour and human papillomavirus exposure to provide a more comprehensive assessment. This will help refine clinical guidelines and ensure the continued safety of transmasculine and gender-diverse individuals undergoing hormone therapy.

## Introduction

Transmasculine and gender diverse (TMGD) people are those individuals whose gender does not align with the female sex that they were assigned at birth.^1^ They may identify as male, non-binary or otherwise gender-nonconforming. Medical treatment of this group may consist of gender-affirming hormone therapy (GAHT), specifically exogenous testosterone, to align physical traits to their gender identity. Despite widespread usage of GAHT in TMGD individuals, data on the long-term histological effects on the reproductive organs are lacking.^2^

Medical transition may also consist of gender affirming surgery (GAS). Historically, removal of the reproductive organs was listed as a required step for legal change of gender in some countries, and remains in others. However, in recent years, growing acceptance of the transgender (trans) community has led to less restrictive laws in many societies.^3^

The abolition of laws that require hysterectomy with bilateral salpingo-oophorectomy for legal change of gender may be one reason for the growing number of TMGD individuals who choose to retain these gynaecological organs or postpone GAS.^4^ Considering the prolonged exposure of gynaecological organs to testosterone, it is important to evaluate the safety and effects of exogenous testosterone on those organs.

The decline in TMGD people undergoing a hysterectomy means that the majority will retain a cervix, and remain at risk for cervical cancer.^4^ Screening based on cervical cytology and high-risk human papillomavirus (hrHPV) testing have shown to be effective strategies to substantially decrease cervical cancer incidence.^5^ However, there are notable barriers with regards to receiving appropriate screening in TMGD individuals. These include psychological discomfort due to triggering gender dysphoria, physical pain (due to atrophy and/or hypertonic pelvic floor), suboptimal patient–clinician relationships and a lack of focus on TMGD individuals in screening programs.^6^^,^^7^ This leads to higher rates of inadequate cervical cytology results and lower rates of receiving regular cervical cancer screening (CCS).^8^^,^^9^ For a detailed description of the screening program in the Netherlands we refer to Supplementary Files.

Higher levels of testosterone can lead to histological changes in the cervix such as squamous atrophy with small basophilic cells or transitional cell metaplasia.^10^ However, it remains unclear whether exogenous testosterone usage is related to developing cervical pathology such as dysplasia or malignancy, and if chances are higher compared to a population of cisgender women (whose gender identity does align with the female sex they were assigned at birth). Nash et al. (2018) reported lower rates of cervical cancer in the trans population compared to cis women in the US.^11^ However, as they were unable to stratify the results according to the sex assigned at birth, these numbers are difficult to interpret, due to lack of an appropriate denominator.^11^ To our knowledge, there have been only two published case reports^12^^,^^13^ of cervical cancer in TMGD individuals on testosterone and a limited number of smaller studies describing cervical intraepithelial neoplasia (CIN) in CCS.^14^, ^15^, ^16^ The majority of CIN lesions were cases of mild dysplasia (CIN1) which regresses in 60–80% of cases, whereas moderate dysplasia (CIN2) and severe dysplasia (CIN3) have elevated risk of progression to cancer and are thus considered “high grade” premalignant lesions.^17^^,^^18^ Importantly, dysphoria and mistrust may cause underreporting of CIN2, CIN3 and cervical cancer.

We here describe cervical histopathology in a large cohort of TMGD individuals using testosterone, compare the incidence of cervical cancer and CIN with the cisgender (cis) female population and determine treatment related risk factors.

## Methods

### Study design and population

For this retrospective cohort study we combined data from the Amsterdam Cohort of Gender Dysphoria (ACOG) with the Nationwide Network and Registry of Histopathology and Cytopathology in the Netherlands (PALGA).^19^ For the ACOG all individuals who visited the gender identity clinic of the Amsterdam UMC between February 17th 1972 and December 31st were identified. In the Netherlands, the vast majority of all transgender individuals seeking gender-affirming care visit our centre for psychological, endocrine or surgical treatment. In this study only individuals who were assigned female at birth and who received testosterone treatment were included. This database was linked to PALGA, a database containing all pathological reports of patients in the Netherlands. Since PALGA covers excerpts of all pathology reports generated since 1991, we also excluded individuals who had their last visit to our clinic before 1991.

The hormone treatment for TMGD individuals generally consisted of testosterone treatment alone. For individuals who were younger than 18 years at start of hormone treatment, testosterone was often preceded by a gonadotropin-releasing hormone agonist. Testosterone preparations prescribed were intramuscular injections testosterone esters or undecanoate (Sustanon® and Nebido®), transdermal testosterone gel (AndroGel® and Tostran®), or undecanoate capsules (Andriol®). In some cases testosterone treatment was combined with progesterone treatment, or a gonadotropin-releasing hormone agonist for prevention of uterine bleeding, or other hormone contraceptives.

This study adheres to the EQUATOR Network's STROBE guidelines for reporting observational studies.^20^ The ethical review board of the VU University Medical Centre Amsterdam concluded that the Medical Research Involving Human Subjects ACT (WMO) did not apply to this study. Necessity for informed consent was waived considering the retrospective design and large study population. All data were processed anonymously.

### Data collection

Data such as medical history, age at start of hormone treatment, type of hormone treatment (including other than testosterone), endocrine laboratory results and data on gender-affirming surgeries were retrieved from medical records. Data were, if available, retrieved from records of visits at start testosterone treatment, and thereafter at two, five and every ten years of follow-up. Data on histopathology obtained from the PALGA database included type of cervical histopathology, histopathological diagnosis (i.e. CIN or cervical cancer [squamous cell carcinoma]), and date of diagnosis.

### Statistical analysis

Cohort characteristics are presented as mean ± standard deviation when normally distributed, median (interquartile range or range) when not normally distributed, or as frequencies and percentages as appropriate. Distribution was assessed using histograms. Only data prior to the (latest) date of surgery or biopsy, (e.g. usage of other hormone treatment) were included in the analysis. Mean testosterone and oestradiol levels were calculated by averaging all measurements during GAHT, excluding the measurement at start GAHT. Mean BMI during treatment was calculated similarly as hormonal levels. Duration of testosterone treatment was calculated from the first known date of start of testosterone until date of histopathological specimen retrieval (biopsy or surgery). In individuals who had started GAHT prior to their first visit at our clinic, the first known start date of GAHT was used to calculate treatment duration.

Follow-up time, which equals testosterone exposure time, was defined as years from start of testosterone treatment until either ≥ CIN2 diagnosis, cervical cancer diagnosis, date of total hysterectomy, date of death or end of the study period (December 31, 2018). In individuals who began treatment before 1991 but had not undergone a hysterectomy by that year (when PALGA initiated national coverage), the follow-up time was set to start on the 1st of January 1991. The sum of all participant's follow-up time was used as total follow-up time (person years). To calculate the age-adjusted standardised incidence ratio (SIR), we used the observed cases and the expected cases of cervical cancer and CIN2 and CIN3 in our cohort. Considering cisgender females are generally diagnosed after screening or testing due to symptoms (e.g. abnormal bleeding), and not after hysterectomy, but TMGD individuals might undergo screening less frequently, two analyses were performed to compare the incidence of CIN in TMGD and cisgender females: the first including all CIN cases in TMGD and the second only including cases from our cohort that were discovered similarly (due to symptoms or abnormal screening). The number of observed cervical malignancies in our cohort were compared to the expected cases based on age-specific incidence rates for cisgender women from the Netherlands Comprehensive Cancer Organisation (IKNL). Since the IKNL also generates cervical cancer incidence rates using data from the same source (PALGA), this allowed for a reliable comparison.

Similarly, age-specific incidence rates of ≥CIN2 were calculated by data obtained from PALGA public database and the Dutch Central Statistical Office (CBS). In the PALGA public database adenocarcinoma in situ (AIS) is classified under CIN3. Subsequently the expected cases of ≥CIN2 in our cohort could be calculated. To calculate 95% confidence intervals normal approximation of the Poisson distribution was used. SIRs with 95% confidence interval (95% CI) were calculated using a mid-exact P test.

Subsequently, we assessed the risk of developing ≥ CIN2 over time within this cohort. Since follow-up time corresponded to the duration of testosterone treatment, we used Kaplan–Meier analysis to evaluate the cumulative risk of CIN2 and CIN3. However, it is important to note that the increasing likelihood of disease detection over time, regardless of treatment, and the role of screening participation may influence these findings. Furthermore, due to lack of data we could not control for individuals who may have discontinued testosterone treatment. Time to event was defined as time from start treatment until CIN2 or CIN3. Cox regression was conducted to evaluate potential risk factors including age at start treatment, oestrogen and testosterone levels, mean BMI during GAHT and smoking. For categorical variables (e.g. smoking), hazard ratios are calculated relative to a baseline category (e.g., non-smokers). For continuous variables (e.g. age or BMI), the hazard ratios represent the effect of a one-unit increase on the risk of developing CIN2 or CIN3. To analyse the (potential) risk to develop CIN2 or CIN3 for individuals who retain their uterus, participants were censored after undergoing hysterectomy. Individuals who had undergone a hysterectomy but of whom no histology results were available, were excluded in both the Kaplan–Meier analysis and Cox regression.

The prevalence of comorbidities linked to cervical dysplasia (i.e. HIV, immunosuppressive medication) was too low for inclusion in the Cox regression, as none of the participants with ≥CIN2 had these conditions. Ethnicity was not included as covariate, as its association with cervical dysplasia is often influenced by socio-economic factors, and 96% of our population was white. To assess the impact of missing data, we compared participants included in the Cox regression (without missing data) to the overall study population. No significant differences were found in age, testosterone use duration, BMI, hormonal levels, or smoking status. This suggests the missing data was random and did not bias or affect the study's results.

STATA Statistical Software, version 17 (Statacorp, College Station, TX, USA) and OpenEpi version3.01 (www.OpenEpi.com) were used for statistical analyses.

### Role of the funding source

There was no funding source for this study. AV, WLJvV, CMW and NMvM had full access to the data. CMW had final responsibility for the decision to submit for publication.

## Results

**Fig. 1:**
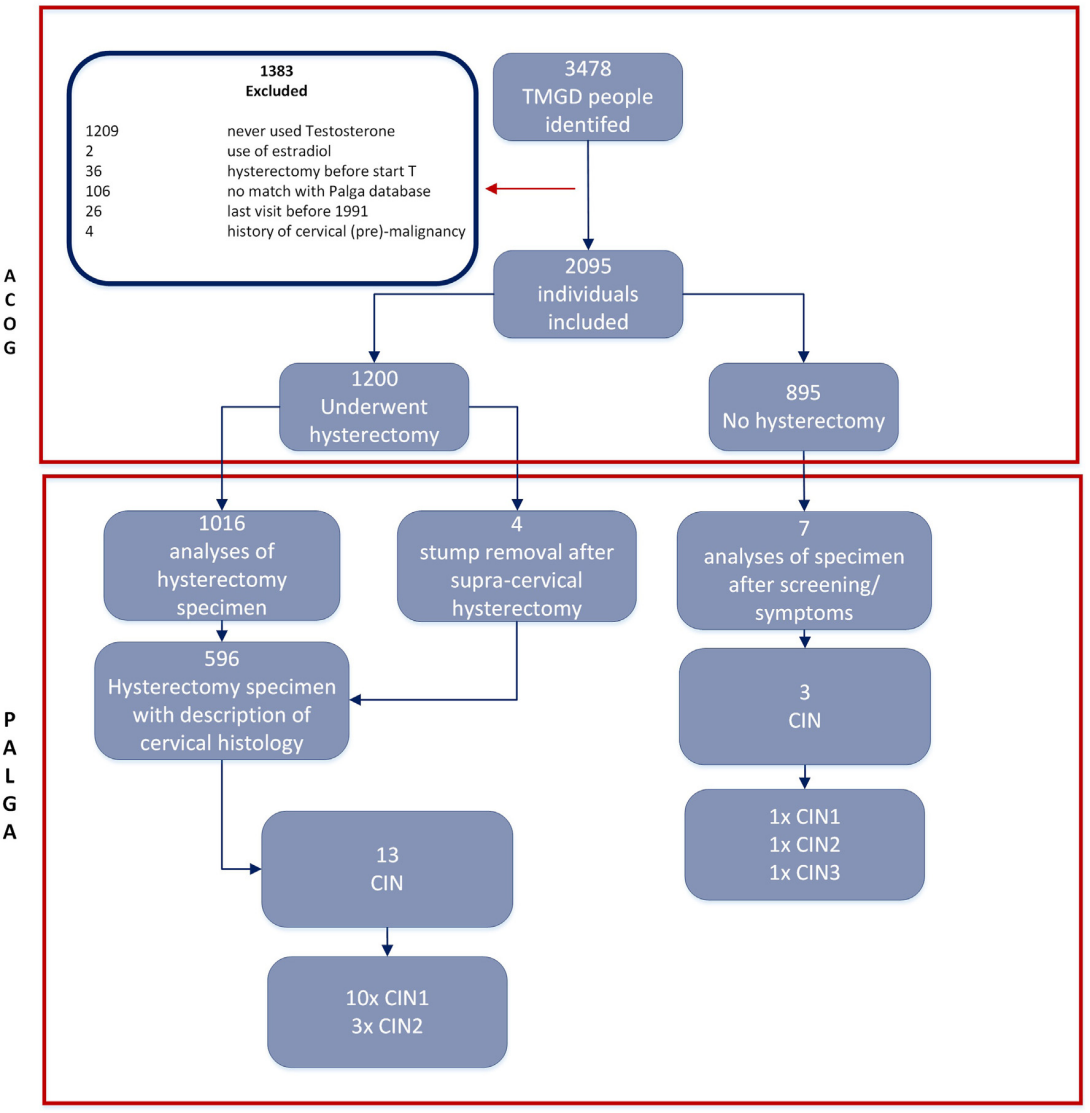
**Study flowchart**. T, testosterone; TMGD, trans masculine and gender diverse; CIN, cervical intraepithelial neoplasia.

The cohort comprised of a total of 3478 TMGD individuals. After exclusion of 1383 people, 2095 individuals were included in the current study (Fig. 1). Table 1 shows an overview of characteristics of the study cohort. The median age at start GAHT was 21 (IQR 18–28) years. Median serum testosterone and oestradiol concentrations were within the female reference range before initiation of GAHT. During GAHT, median testosterone concentrations were within therapeutic range (20.5 nmol/L (IQR 13–31.9)). Median oestradiol levels during GAHT were 111 pmol/L (71–166). The total follow-up time, which equals testosterone exposure time, ranged from 0.003 to 28.0 years, with a median of 1.6 years (IQR 1.3–2.2). In 98% of participants (2045/2095), the follow-up time equalled the duration of testosterone exposure. In 45 participants, the minimum follow-up time was applied, starting on the 1st of 1991. In total 6.4% (134/2095) individuals had an exposure exceeding 5 years. The median age at time of hysterectomy was 25 (IQR 21–33).

**Table 1:** Study cohort characteristics.

	N (total)^a^	Median (IQR) unless stated otherwise
Age at start GAHT, years	2095	21 (18–28)
BMI during treatment (mean)^b^	1941	23.9 (21.5–27.5)
Serum testosterone concentration before initiation of GAHT^c^, nmol/L^a^^,^^d^	891	1.3 (1.0–1.6)
Serum testosterone concentration during GAHT (mean), nmol/L^b^^,^^d^	1414	20.5 (13.0–32.0)
Serum oestradiol concentration before initiation of GAHT, pmol/L^a^^,^^d^	903	170 (59–405)
Serum oestradiol concentration during GAHT, pmol/L^a^^,^^b^	1371	120.1 (78–179)
Age at time of hysterectomy	1200	25 (21–33)
Total follow-up (years) (Testosterone exposure)		
Total cohort	2095	1.7 (1.3–2.5) min 0.003 max 28.0 (134 [6.4%] > 5 years)
Hysterectomy group	1200	1.6 (1.3–2.3) min 0.003 max 15.0 (44 [3.7%] > 5 years)
No hysterectomy group	895	1.8 (1.2–3.0) min 0.003 max 28.0 (90 [10.1%] > 5 years)
(former) smokers	1313	812 (61.9%)
Known use of immunosuppressive medication	2090	9 (0.4%)
HIV+	2090	3 (0.1%)

BMI during treatment (mean): 92.6% of data available.

Serum T before initiation of GAHT: 45.9% of data available.

Serum T during treatment (mean): 67.5% of data available.

Serum E before initiation of GAHT: 46.5% of data available.

Serum E during treatment (mean): 65.4% of data available.

Smoking: 62.7% of data available.

a Excluding missing.

b Mean of all measurements taken at follow-up appointments during treatment (at two, five and every ten years of follow-up).

c GAHT, gender-affirming hormone treatment.

d Individuals with a start date prior to their first visit at our clinic were excluded from analysis (n = 151).

Fig. 1 shows an overview of the type of histopathology specimens that were included in the study. In total, 1200 individuals underwent hysterectomy of whom 1016 pathology results were available of which 596 included a description of cervical histology. Additionally, 4 cases of cervical stump removal were identified in patients who had undergone a supra-cervical hysterectomy. Seven individuals had biopsies taken because of symptoms or abnormal pap-smear screening.

### Cervical cancer and ≥CIN2 in TMGD compared to cisgender population

No cases of cervical cancer were observed in the study cohort. Based on age-specific incidence rates in the cisgender population, the number of expected cervical cancer cases in our cohort would be 0.30 (95% CI 0–1.4). Since no cases of cervical cancer were observed in our cohort a standardised incidence ratio could not be calculated.

In total, 11 cases of CIN1 (0.005%), 4 cases of CIN2 (0.002%) and one case of CIN3 (0.0005%) were observed. Median duration of testosterone at time of diagnosis of ≥CIN2 was 1.8 years (range 0.1–2.8). No cases of AIS or other cylindric abnormalities were found.

**Fig. 2:**
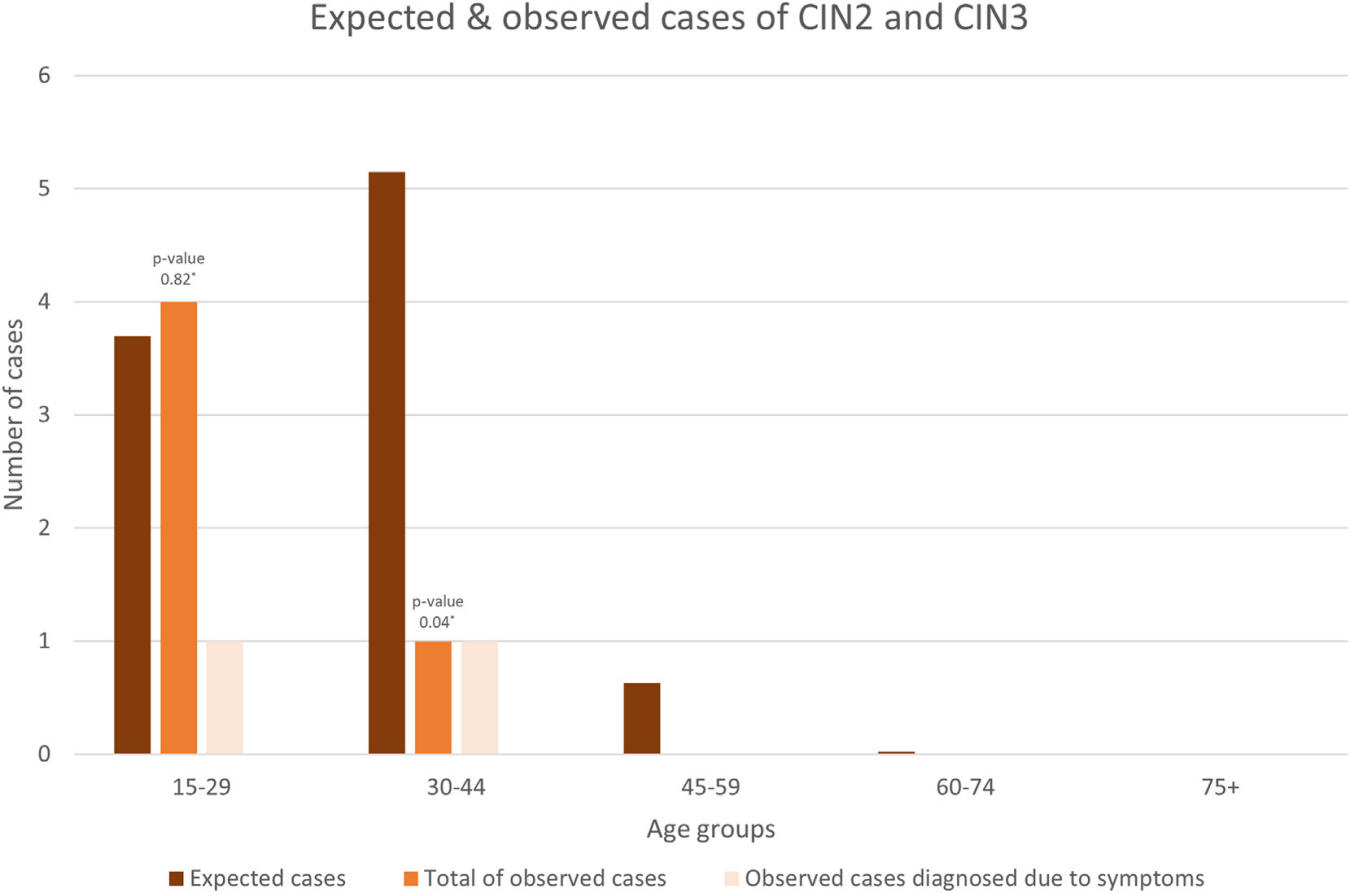
**Expected versus observed cases of≥CIN2**. ∗Mid-p exact test.

Based on the incidence of ≥CIN2 in the cisgender population the number of expected cases of ≥CIN2 would be 9.5 in our cohort. In total 5 cases of ≥CIN2 (0.002%) were found, two of which were detected after an abnormal Pap smear (see Fig. 2). When comparing all observed cases to the CIN population this resulted in a SIR of 0.53 (95% CI 0.19–1.17). The subanalysis only including the two cases diagnosed by biopsy specimen resulted in a SIR of 0.21 with a 95% confidence interval of (0.04–0.70).

### Accumulated risk and risk factors of ≥CIN2 within TMGD

**Fig. 3:**
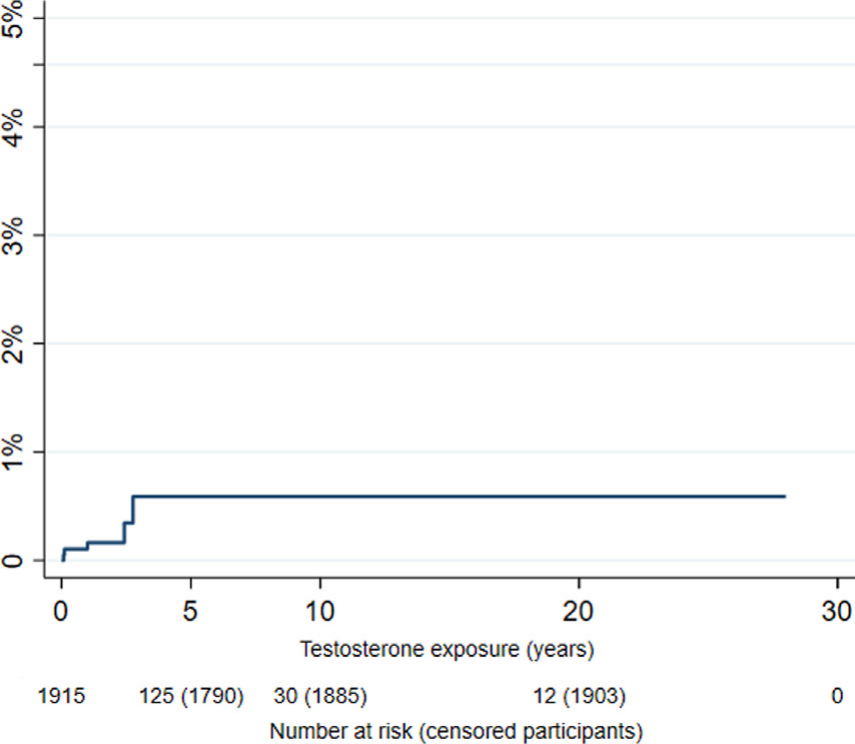
**Kaplan–Meier curve displaying the risk of ≥CIN2 for individuals who retain their uterus**. All 5 events (≥CIN2) occurred within 3 years of testosterone use, resulting in a steady risk after 3 years of treatment. Additionally, as the majority undergoes hysterectomy before 5 years of testosterone treatment the risk set declines rapidly.

The Kaplan–Meier analysis showed a risk of 0.59% (95% CI 0.21–1.69%) to develop ≥ CIN2 after 5 years of treatment for individuals who retain their uterus (Fig. 3). There was no evidence that age at start testosterone treatment (hazard ratio (HR) 1.02 [95% CI 0.93–1.11]), testosterone (HR 0.94 [95% CI 0.84–1.06]), oestradiol levels (HR 1.0 [95% CI 0.99–1.01]), mean BMI (HR 1.08 [95% CI 0.93–1.25]) or smoking (HR 2.02 [95% CI 0.21–19.57]) were associated with an increase or decrease of this risk. A risk table is included in the Supplementary Files.

## Discussion

The aim of this large cohort study was to assess the risk of cervical (pre)malignancies in TMGD individuals using testosterone compared to a cisgender female population. No cases of cervical cancer were observed. In total 16 cases of cervical intraepithelial neoplasia were diagnosed, of which 4 cases of CIN2 and one case of CIN3. We found no increased risk of cervical cancer or ≥ CIN2 in individuals using testosterone compared to cisgender women. Based on our observations the risk to develop ≥ CIN2 after 5 years of treatment for individuals who retain their uterus is 0.59% (95% CI 0.21–1.69%).

Given the necessity to undergo hysterectomy for legal gender change in the Netherlands until 2014, this cohort is young, with a short time of hormone therapy. The majority of individuals undergo hysterectomy before they reach the age at which the incidence of cervical dysplasia and cancer peaks. Whereas rates of hysterectomy for TMGD in the Netherlands pre-2014 were around 84%,^21^ a fall in rates may be seen as a result of changing attitudes. Rates are also likely to be far lower where there is limited access to this surgery via public healthcare, with rates as low as 14% in the US.^4^ Therefore, it is difficult to comment on the long term effects of testosterone (and any oestradiol suppression) on CIN or cervical cancer development.

Increased serum testosterone levels have been linked to both CIN and cervical cancer in cisgender women, but this has been postulated to be related to increased bioavailability of oestrogens.^22^^,^^23^ Oestrogen exposure has also been linked to cervical cancer development,^23^^,^^24^ and is posited to cooperate with HPV infection to drive progression from premalignant to malignant disease.^24^

Crucially, in TMGD individuals on GAHT much higher serum testosterone levels are achieved, and although serum oestradiol is repressed to some degree by negative feedback, aromatisation of testosterone to oestrogen does still occur and oestrogen levels are not much lower. This is evident in our cohort. However, we are unable to comment on prior oestrogen exposure (e.g. age at menarche and the use of oestrogen containing contraceptives) which is also likely to be of biological relevance. Nevertheless, in this cohort neither testosterone nor oestradiol levels were associated with an increased risk of ≥CIN2. However, the statistical (in)significance should be interpreted with caution considering the small number of participants in ≥CIN2 group.

Persistent high-risk HPV (hrHPV) is the major causative agent in cervical cancer development. In the Netherlands, primary hrHPV testing replaced cytology screening in the National Screening Programme in 2017 and so HPV status was only available for a subset of participants.^25^ hrHPV is sexually transmitted and risk of acquisition, persistence and subsequent CIN and cancer are linked to sexual behaviour. Importantly, risk factors such as the risk of hrHPV acquisition may differ significantly between TMGD individuals and cisgender women, complicating the comparison. Dysphoria may be a cause for transgender individuals to withhold from sexual activities before gender-affirming surgery, potentially leading to lower risk of hrHPV exposure compared to their peers. However, this information on sexual behaviour is not available for this cohort, and heterogeneity within the TMGD community in sexual behaviour may mask effects. Studies have shown prevalence of hrHPV (as measured by cervical/vaginal swabs and urine) in transgender men to range from 10.2 to 28%, with a tendency to be similar to that of cisgender women in the country of study.^26^^,^^27^ Studies have shown a relationship between anal HPV-persistence and free testosterone in men who have sex with men,^26^ but the mechanism for this remains unclear. The same has not been shown for cervical HPV. In cisgender women, a vaginal microbiome deficient of lactobacilli has been associated with increased risk of vaginal infections, including persistent HPV infection.^28^ Some studies have reported that TMGD individuals using testosterone are more likely to have vaginal flora enriched with other bacterial species than lactobacilli.^5^ However, these studies did not compare microbiota pre- and post-initiation of testosterone treatment.

National cervical screening in the Netherlands begins at age 30.^25^ It is reassuring that the average age of CIN abnormalities was significantly older than for those without, but fell under the age at which screening begins. This is not unexpected considering the vast majority of patients in our cohort underwent a hysterectomy before the age of 30. Notably, the majority of CIN lesions were CIN1, which regresses in 80% of cases.^18^ It is difficult to say if any of the CIN1 cases would have progressed to CIN2 or CIN3, or invasive disease if untreated. CIN2 and CIN3 lesions were detected at an average age of 28. Given the significantly lower age-adjusted incidence observed in our population compared to the cisgender population, there is insufficient justification to initiate screening at a younger age for transgender and gender diverse individuals. Importantly, irrespective of age, screening is always indicated in case of abnormal bleeding.

TMGD individuals face multiple barriers when engaging with conventional cervical screening, including discomfort from speculum exams (due to vaginal atrophy on testosterone), dysphoria from the procedure, cisnormative environment or available information, lack of awareness risk, lack of ability to access screening recall due to a male gender marker and anticipated or experienced discrimination.^29^ Screening engagement for those who retain their uterus depends on reducing these barriers. hrHPV self-sampling, either by vaginal swab or first void urine, has been shown to be highly acceptable in this population.^27^ Recent organ-based screening recommendations for sexual and gender minorities in the US recommend “Screen all individuals with cervices. Self-collected vaginal swabs should be recommended to patients as an option for sample collection once approved”.^30^

Other studies reported cervical atrophy and cervical metaplasia in TMGD individuals using testosterone.^10^ Considering atrophy increases the risk of inadequate sampling and metaplasia can mimic CIN2 or CIN3 at colposcopy,^30^ primary hrHPV testing and self-sampling are crucial in reducing the numbers of TMGD individuals requiring repeat testing or unnecessary biopsies. Recently, the effectiveness of bivalent HPV vaccination in preventing invasive cervical cancer was confirmed.^31^ However, TMGD may be less likely to be vaccinated against hrHPV than cisgender individuals.^32^ Improving vaccination rates amongst transgender teens by lowering barriers that might prevent them to participate in vaccination programs is therefore paramount.^32^

One of the major strengths of this study is its large cohort size. Additionally, our data validation by linkage to the PALGA database, which offers nationwide coverage, enhances the reliability of our findings. We believe this is the first study to provide a robust estimation of the risk of cervical cancer and high-grade dysplasia in TMGD individuals.

In addition, there are several limitations to consider when interpreting these results. Firstly, there is the potential for information and verification bias given that TMGD individuals are more likely to undergo hysterectomy at a young age than cisgender women, potentially leading to relative overreporting of pathology. Conversely, TMGD individuals may be less likely to participate in regular screening, and less likely to have a biopsy collected if indicated after screening. This could result in underreporting or diagnosis at more advanced stages. As these two factors likely counterbalance each other, and as we performed a subanalysis only including the cases diagnosed after abnormal screening or symptoms, we do not anticipate a significant impact on the overall reliability of our results. Another limitation in our cumulative risk analysis is the lack of data on individuals who may have discontinued testosterone treatment, as we were unable to censor or control for this. However, a recently published review reported five studies in which over 90% of individuals initiating gender-affirming hormone therapy continue it long-term. Therefore, we believe this limitation has minimal impact on our findings.^33^ Additionally, in this cumulative risk analysis, showing a limited risk increase related to longer testosterone exposure, other potential risk factors such as sexual behaviour and HPV exposure were not adjusted for as this information was not available. Furthermore, we could not adjust for these or other confounding factors in the comparison with the cisgender population, as we only had access to incidence rates for this group. These data should be collected in any future prospective studies. Notably, the authors of this study underline that considering these risk factors could not be taken into account and the major risk of TMGD individuals who retain their uterus with regard to cervical cancer lies in barriers to screening at multiple levels, efforts to reduce these, and encourage screening and vaccine uptake, should persist.

In conclusion, we found no increased risk of invasive cervical cancer or high grade CIN in TMGD using gender affirming hormone therapy, though effects could be masked by the young age of hysterectomy and relative short time of testosterone use in this cohort. HPV cervical screening remains indicated as routine care for all TMGD with a uterus.

## Contributors

AV, CW, and NvM contributed to the study design and data collection. AV, WvV, AB, CW, and NvM were involved in data analysis. AV, WvV, and AB drafted the original manuscript. MS, MdH, FG, JH, CW, and NvM reviewed and edited the manuscript. AV, WvV, NvM, and CW had access to and verified the underlying study data. All authors critically revised the manuscript, contributed to the data interpretation, and had final responsibility for the decision to submit for publication. CW and NvM share last authorship.

## Data sharing statement

Statistics Netherlands prohibit data sharing at an individual level to guarantee the anonymity of the people in its databases.

## Declaration of interests

All authors hereby attest that they do not have any relevant conflict of interest related to this article.
