# Supplementary material for: Incidence of cervical intraepithelial neoplasia and cervical cancer in transmasculine and gender diverse individuals using testosterone: a retrospective, single-centre cohort study
Source: eClinicalMedicine. 2025 Jan 13;80:103037. doi: 10.1016/j.eclinm.2024.103037 (PMC11934862; doi:10.1016/j.eclinm.2024.103037)
Supplement: Supplementary Data [file mmc1.pdf]

Description Dutch screening program for cervical cancer

In the Netherlands cisgender women aged 30 to 60 are invited to schedule a screening appointment with their primary care doctor every five years. In 2017, the program switched from cytomorphological to primary high-risk HPV(hrHPV) screening. Individuals with a cervix who are registered as man or X/O can apply to participate in the screening program.
If hrHPV is detected during screening, a Pap smear is performed. Individuals with a hrHPV positive ánd ≥pap 2 test result are referred to a gynaecologist. If the Pap smear result is Pap 1, it is repeated in 12 months.

1. Aitken CA, van Agt HME, Siebers AG, et al. Introduction of primary screening using high-risk HPV DNA detection in the Dutch cervical cancer screening programme: a population-based cohort study. *BMC Medicine* 2019; **17**(1): 228.

Risk table Cox-regression

Covariates	Participants*	Hazard Ratio	p-value	95% conf. interval
**Age at start GAHT**	n=1915	1·02	0·70	0·93 – 1·11
**Testosterone levels**	n=1281	0·94	0·32	0·93 - 1·11
**Oestrodial levels**	n=1242	1·0	0·79	0·99 – 1·01
**Mean BMI**	n=1792	1·08	0·32	0·93 – 1.25
**Smoking**	n=1204	2·02	0·54	0·21 – 19·57

* Excluding missing (total participants: n= 1915)
